# Potential Application of *Ixeris dentata* in the Prevention and Treatment of Aging-Induced Dry Mouth

**DOI:** 10.3390/nu10121989

**Published:** 2018-12-15

**Authors:** Kashi Raj Bhattarai, Hwa-Young Lee, Seung-Hyun Kim, Jong-Sug Park, Hyung-Ryong Kim, Han-Jung Chae

**Affiliations:** 1 Department of Pharmacology and Institute of New Drug Development, Chonbuk National University Medical School, Chonbuk, Jeonju 54896, Korea; meekasik@jbnu.ac.kr (K.R.B.); youngat84@gmail.com (H.-Y.L.); 2 College of Pharmacy, Yonsei Institute of Pharmaceutical Science, Yonsei University, Incheon 406-840, Korea; kimsh11@yonsei.ac.kr; 3 Department of Agricultural Biotechnology, National Institute of Agricultural Science, Jeonju 54875, Korea; jongsug@korea.kr; 4 Daegu Gyeongbuk Institute of Science and Technology (DGIST), Daegu 42988, Korea

**Keywords:** aging, hyposalivation, dry mouth, ER stress, *Ixeris dentata*, salivary gland

## Abstract

Dry mouth is a common complaint among the elderly population. The aim of this study was to investigate the effect of Ixeris dentata (IXD) extract on aging-induced dry mouth. We used young (two months) and aged (20 months) SD rats in our study. Using water as the vehicle, IXD extract (25, 50, and 100 mg/kg) was given via oral gavage to the young and aged rats for eight weeks. We found that the salivary flow rate relative to the submandibular gland weight was differently influenced by IXD extract treatment. IXD extract augmented the submandibular gland acinar cells, which are depleted during aging. In addition, the decreased salivary alpha-amylase, inositol triphosphate receptor, and aquaporin-5 in the aging rats were upregulated by IXD treatment. Free radical-induced oxidative stress in the aging rats was also alleviated in the IXD-treated group. The formation of high molecular weight complexes of protein disulfide isomerase, decreased expression of an ER chaperone (GRP78), and increased ER stress response (ATF-4, CHOP and p-JNK) in aging rats was regulated with IXD treatment, and eventually increased salivary secretions from the aging submandibular glands. These are the first data to suggest that IXD extract might ameliorate aging-associated oral dryness by regulating the ER environment.

## 1. Introduction

Saliva plays an important role in maintaining oral health; however, salivary secretions and their constituents alter with age. More than 30% of the elderly population suffers from xerostomia and related complications [1,2]. One mechanism implicates a reduced glutathione and oxidized glutathione ratio (GSH: GSSG) in that process [3,4]. The free-radical theory of aging, including oxidative stress, has received much attention from researchers. A previous study showed that the unstimulated salivary flow rate decreases with age because of physiological changes during the aging process or atrophy in the salivary glands [3]. Another study reported that the cause of dry mouth syndrome in older people may be due to the consumption of xerogenic drugs such as antihistamines, antihypertensive, antidepressants, and antipsychotics during age-related medical treatment [5,6,7]. Hyposalivation is a major oral health problem in the elderly, resulting in salivary gland dysfunction. The complications of aging include xerostomia, periodontitis, bone loss, dental caries, tooth demineralization, dysphagia, burning mouth sensation, and taste disorders [8,9]. In addition, cellular secretory functions and the activities of the salivary glands both decline with age. Aging alters the salivary levels of sodium, potassium, proline-rich proteins, lactoferrin, lysozyme, mucins, sIgA, total protein, transforming growth factor-alpha, and salivary alpha-amylase [6,10]. Several proteomic tools have been discovered to identify biological markers [11], however to provide better information about disease progression, point of care (PoC) test for the detection of active matrix metalloproteinase-8 in periodontal disease is suggested [12]. Age is one of the most influential factors in oxidative stress and protein carbonylation, which have been suggested as biomarkers of aging. If saliva secretion decreases as part of aging, the salivary antioxidant system also declines, which can increase the presence of reactive oxygen species and reactive nitrogen species [13]. The high production of free radicals promotes high-level oxidative stress, which eventually damages cells and is associated with endoplasmic reticulum (ER) stress [14].

The ER is an important cellular organelle involved in protein folding, maturation, and homeostasis [15]. Several chaperones and foldases that take part in folding and quality control, such as GRP78, GRP94, calnexin, calreticulin, ERP57, and PDI, reside in the ER at high concentrations [16,17]. The ER quality control machinery helps to reduce protein aggregation, ensuring that transcription and translation occur correctly by chaperoning unfolded proteins or selectively degrading improperly folded polypeptides before they can aggregate [16,18]. However, the potency of those components declines during aging [19,20].

Recently, we used a diabetic rat model to identify *Ixeris dentata* (IXD, Korean name Sseumbagwi) as a regulator of salivary amylase secretion [21]. Many studies have considered the components and nutritional value of IXD [22]. Our previous study reported the identification of several compounds in the IXD extracts extracted from ethanol. Among many, Ixerin F, Ixerin M, and 8-epiisolipidiol-3-β-d-glucopyranoside (8-EI-3-G) have a greater capacity to secrete alpha-amylase from the salivary gland cells [21]. Similar to other flavonoid-enriched natural extracts, the antioxidant effects of IXD have been confirmed. However, its specific functions have not yet been studied in conditions such as aging. Many elderly people in countries such as Korea, Japan, and China already use IXD extract, especially for dry mouth symptoms. In those countries, IXD extract is popular and is widely understood to be a functional or healthy food [21,23]. 

It is still unknown how the aging alters the salivary dysfunction. We hypothesized that the disruption in the ER folding capacitance impairs the maturation and folding of amylase or aquaporin-5, might be a possible mechanism of aging-associated dry mouth. The aim of our study was to investigate the cellular mechanisms that influence salivary gland during aging, its consequences, and possible prophylactic action. The null hypothesis of our study was that, in the aging process, there would be no difference in the salivary flow rate and the suggested mechanisms even in the IXD-treated condition.

## 2. Materials and Methods 

### 2.1. Plant Material Preparation

*Ixeris dentata* root samples were collected in October 2015 and identified by Dr. Sang-Won Lee, a senior researcher in the National Institute of Horticultural and Herbal Science, Rural Development Administration, Korea (RDA). Pulverized, dried roots (10 g) were extracted three times in an ultrasonicator with 50 mL of 100% ethanol for six hours. They were then filtered using Whatman filter paper No. 1 (Maidstone, UK) and evaporated *in vacuo*. The extract was dissolved in 50% methanol at a concentration of 100 mg/mL and filtered through a 0.45 µm PTFE syringe filter for analysis. The reference standards of Ixerin F and 8-EI-3-G were isolated and purified from *I. dentata* using various chromatographic techniques. For isolation, dried roots of *I. dentata* (5.0 kg) were extracted with MeOH (3 × 10 L, 50 °C) under sonication for 4 h to yield 700.0 g of extract after evaporation of the solvent. This extract was suspended in H_2_O and successively partitioned with CHCl_3_ and EtOAc to obtain CHCl_3_, EtOAc, and H_2_O extracts after removal of the solvents *in vacuo*. The H_2_O fraction was chromatographed on a Diaion HP-20P column eluting with 0, 25, 50, 75, and 100% MeOH to obtain five sub-fractions, ID2A, ID2B, ID2C, ID2D, and ID2E, respectively. The ID2C fraction was chromatographed on HPLC using a J’sphere ODS H-80 250 mm × 20 mm column, 15% aq. MeCN, and a flow rate of 3 mL/min to yield Ixerin F (12.1 mg). The ID2D fraction was chromatographed on HPLC using a J’sphere ODS H-80 250 mm × 20 mm column, 15% aq. MeCN, and a flow rate of 5 mL/min to yield 8-epiisolipidiol-3-β-d-glucopyranoside (10.3 mg). Comparison of the NMR and MS data with values reported in the literature led to the identification of these structures, with purities determined by HPLC–DAD analysis to be >98% based on the peak area normalization method. Individual compounds were prepared at a concentration of 0.5 mg/mL in 100% methanol for standard solutions. A standard mixture containing 0.5 mg/mL of each compound was mixed with 100% methanol to appropriate concentrations by serial dilution.

### 2.2. Quantitation of Pure Compounds Using HPLC-DAD

**Figure A1:**
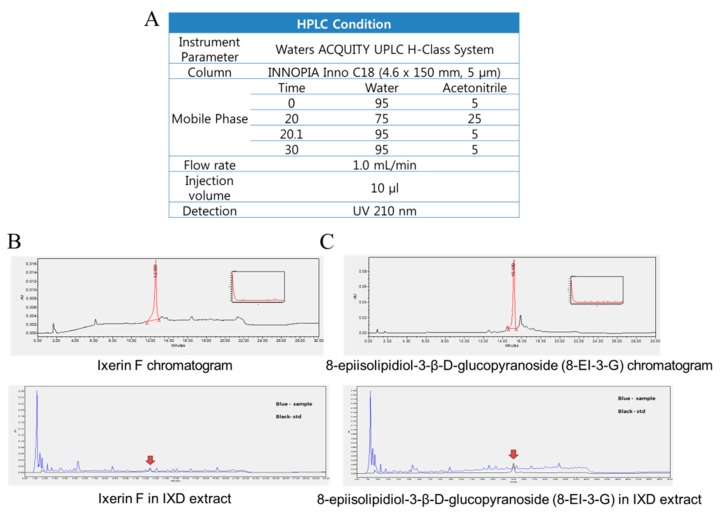
(**A**) HPLC conditions used in the quantitation of pure compounds from the IXD extract; (**B**) Chromatogram of Ixerin F; (**C**) Chromatogram of epiisolipidiol-3-β-d-glucopyranoside (8-EI-3-G).

Chromatographic analyses were performed using a Waters ACQUITY UPLC H-Class System (Waters Corporation, Milford, MA, USA). Chromatographic separation was carried out at 30 °C on an INNOPIA Inno C18 (4.6 × 150 mm, 5 μm). The mobile phase consisted of water (A) and acetonitrile (B) using a gradient elution of 5–25% B at 0–20 min and 5% B for 10 min. The flow rate was kept at 1 mL/min. The injection volume was 10 µL. Different mobile phases (methanol-water, acetonitrile-water, methanol–acid aqueous solution, and acetonitrile-acid aqueous solution) were examined and compared to develop the chromatographic analysis. An eluting solvent system of acetonitrile-water was chosen to provide acceptable separation within a run time of 30 min. The retention times of Ixerin F and 8-epiisolipidiol-3-β-d-glucopyranoside were 12.59 and 15.19, respectively. The HPLC method was validated in terms of linearity using the International Conference on Harmonization guidelines. Six-point calibration curves for each compound were analyzed in triplicate, and acceptable linear correlation and high sensitivity at those conditions were confirmed by the correlation coefficients (*r*^2^, 0.9997–1.0000). The HPLC conditions and chromatograms of Ixerin F and 8-EI-3-G are shown in Figure A1A–C (Appendix A).

### 2.3. Animals and Experimental Designs

**Figure A2:**
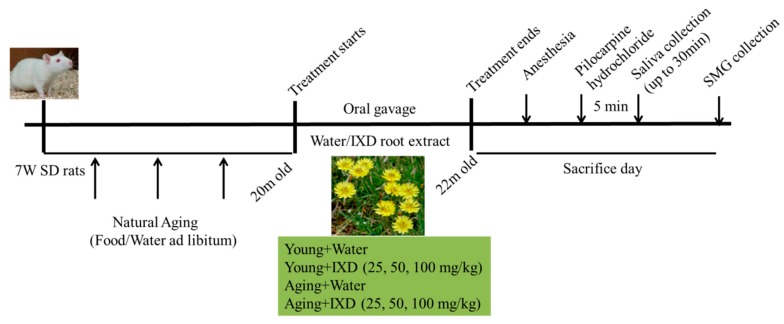
Schematic experimental design and dosage regimen.

In this study, we used 85 adult male SD rats, which we purchased from Samtako Bio Korea. The rats were housed under standard living conditions (22 ± 2 °C, 55–60% relative humidity, and a 12-h light/dark cycle). The rats were acclimatized to our laboratory conditions for one week before use in the experiments. All the animal use protocols were approved by the Institutional Animal Care and Use Committee of Chonbuk National University Laboratory Animal Center (CUH-IACUC-2016-3). The schematic design of our experiment is shown in Figure A2.

### 2.4. Dosage Regimen

The animals were randomly divided into eight groups for the young and aging models (*n* = 10–15 per group). The mortality rate of the aging rats was almost 25%. A diet of tap water and standard food pellets was available *ad libitum* throughout the experimental period. The naturally aging rats (20 months old) and young rats (2 months old) received either water or IXD extract treatment via oral gavage for eight weeks. The animal groups and doses of IXD extract were categorized as: Young group (Water, IXD 25, 50, 100 mg/kg) and Aging group (Water, IXD 25, 50, 100 mg/kg).

### 2.5. Collection of Saliva and Measurement of Salivary Flow Rate 

After eight weeks of water or IXD extract treatment, the rats were anesthetized (ketamine, 1 mL/kg). Pilocarpine hydrochloride (0.6 mg/kg) was injected intraperitoneally to stimulate salivary secretion. Five minutes after injection, pre-weighed small cotton balls were placed under the tongue for 30 min to collect the whole saliva. Cotton balls were changed and maintained on ice for 5, 10, 20, and 30 min to prevent the loss of saliva. To prevent moisture loss, the cotton balls soaked with saliva were weighed immediately using an electric balance. The total saliva was calculated as the difference between the pre-weighed and post-collection cotton balls. The salivary volume was determined gravimetrically, considering a density of 1.0 g/mL for saliva [24]. The salivary flow rate was calculated by converting the total saliva secretion (in microliters) per minute. The data with regard to salivary flow rate presented in our current study was normalized with the weight of submandibular glands.

### 2.6. Amylase Activity Measurement 

Salivary amylase activity was determined using a commercial kit (K711-100, Bio Vision Inc., Milpitas, CA, USA) following the manufacturer’s assay protocol.

### 2.7. Histological Analysis

For the morphological study, routinely processed paraffin-embedded submandibular gland (SMG) tissue was stained by hematoxylin and eosin as described previously [25].

### 2.8. ROS Detection

Intracellular reactive oxygen species (ROS) was detected with dihydroethidium (DHE) (Invitrogen, Molecular Probes). DHE (5 μM) was added to the submandibular gland sections and incubated in the dark for 30 min at 37 °C. The images were obtained using tetramethylrhodamine isothiocyanate (TRITC) channels in confocal microscopy.

### 2.9. Immunoblotting 

Immunoblotting was performed using submandibular gland homogenates as described previously [21] to determine the expression of alpha-amylase, PDI, GRP78, ATF-4, CHOP, sXBP-1, p-JNK, and JNK. The antibodies used for western blotting analysis were anti-amylase (1:1000, anti-mouse, Santa-Cruz Biotechnology, Dallas, TX, USA), anti-PDI (1:1000, clone 1D3, Enzo Life Sciences, Farmingdale, NY, USA), anti-GRP78 (1:2000, anti-mouse, Santa-Cruz Biotechnology, Dallas, TX, USA), anti-CHOP (1:2000, anti-mouse, Cell Signaling, MA, USA), anti-XBP1 (1:1000, anti-mouse, Santa-Cruz Biotechnology, Dallas, TX, USA), anti-p-JNK (1:1000, anti-rabbit, Cell Signaling, MA, USA), JNK (1:1000, anti-rabbit, Cell Signaling, MA, USA), anti-ATF-4 (1:1000, anti-rabbit, Cell Signaling, MA, USA), and anti-actin (1:5000, anti-rabbit, Cell Signaling, MA, USA).

### 2.10. Immunohistochemistry and Immunofluorescence

Immunohistochemistry was performed in formalin-fixed, paraffin-embedded submandibular gland tissue as described previously [21]. Antibodies analyzed with immunohistochemistry were alpha-amylase (1:200, anti-mouse, Santa-Cruz Biotechnology, Dallas, TX, USA), aquaporin-5 (1:150, anti-rabbit, Abcam, Cambridge, UK), 4-Hydroxy-2-nonenal (4-HNE) (1:100, anti-rabbit, Abcam, Cambridge, UK), GRP78 (1:100, anti-mouse, Santa-Cruz Biotechnology, Dallas, TX, USA), and GADD153/CHOP (1:100, anti-rabbit, Santa-Cruz Biotechnology, Dallas, TX, USA). Double-label immunofluorescence was performed using anti-goat IP3R2 (1:100, Santa-Cruz Biotechnology, Dallas, TX, USA) and anti-rabbit AQP5 antibody (1:300, Abcam, Cambridge, UK). Sections were incubated with primary antibodies in a humidified chamber overnight at 4 °C. Slides were then rinsed in 1×TBST buffer (DAKO) three times and incubated with fluorescein isothiocyanate (FITC)-conjugated anti-goat secondary antibody (1:300, Sigma) or TRITC-conjugated anti-rabbit secondary antibody (1:300), respectively, for 1 h at room temperature. Slides were then followed by washing with TBST (3 times) and mounted with DAPI (Invitrogen, Thermo Fisher Scientific, Waltham, MA, USA). Fluorescence was visualized using FITC and TRITC channels in a laser scanning confocal microscope (Olympus, Japan).

### 2.11. Detection of Carbonylated PDI

High molecular weight complex (HMWC) protein disulfide isomerase (PDI) was evaluated in rat submandibular gland lysates by following a previously described protocol [26].

### 2.12. Real-time PCR 

The mRNA expression of Aquaporin-5 (AQP5) and Sodium-hydrogen exchanger (NHE1) was determined by quantitative real-time PCR as previously described [27]. Briefly, total RNA was isolated from submandibular gland tissue using Trizol reagent (Invitrogen Life Technologies, Carlsbad, CA, USA), and complementary DNA (cDNA) was synthesized from the RNA using a PrimeScript Reverse Transcriptase kit following the manufacturer’s protocols. PCR was performed using SYBR Green PCR Master Mix (Applied Biosystems, Foster City, CA, USA). The following rat primers were used in this study: AQP5: Forward: GGGATCTACTTCACCGGCTG, Reverse: AGAGAGGAGGGGAAGAGCAG. NHE1: Forward: TAAGGTGGTCTGGCATCTGG, Reverse, CATCCCCAATTGAGCGTTCC. The target gene mRNA expression level was calculated using the ΔCt method and normalized to actin mRNA expression.

### 2.13. Statistical Analysis

Data are presented as the mean ± standard error of the mean (SEM). The statistical software Origin (Origin-Lab Corporation, Northampton, MA, USA) was used for data analysis. Statistical analyses were performed using a one-way analysis of variance, and Tukey’s test was done for the individual means. A *p* value < 0.05 was set as the criterion for statistical significance.

## 3. Results

### 3.1. Ixeris dentata Improves Aging-Induced Salivary Dysfunction

**Figure 1:**
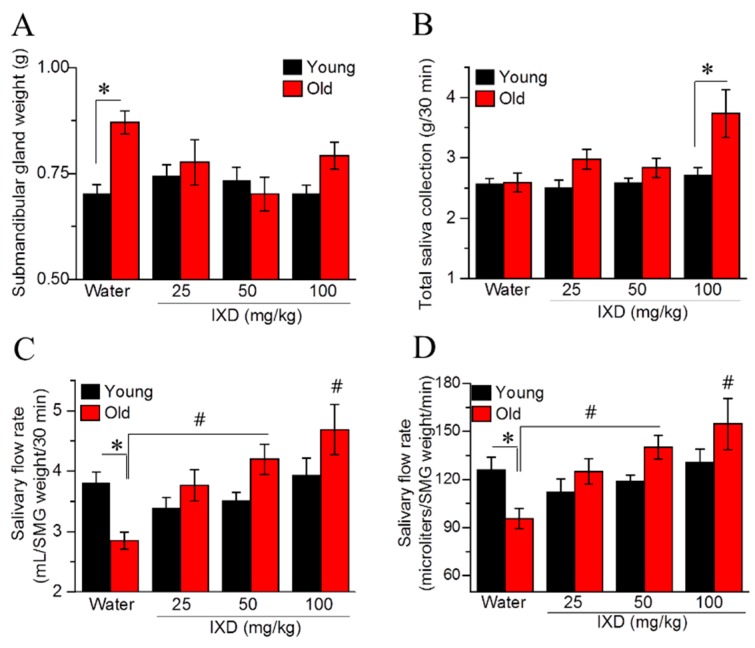
Ixeris dentate (IXD) improves aging-induced salivary dysfunction. Water or IXD extract was given orally to young and aging rats for eight weeks. Saliva and the submandibular glands were collected on the sacrificing day. (**A**) Weight of submandibular glands (g), * *p* < 0.05 vs. young control rats; (**B**) Total saliva collected over 30 min, * *p* < 0.05 vs. young IXD 100 mg/kg group; (**C**) Salivary flow rate by submandibular gland weight (mL/SMG weight/30 min); (**D**) Salivary flow rate by submandibular gland weight per minute (µL/SMG weight/min). * *p* < 0.05 vs. young control rats, # *p* < 0.05 vs. old control rats.

We found that the submandibular gland weight of aging rats was significantly increased compared with that from young rats (*p* < 0.05) (Figure 1A). We observed no differences in whole saliva volume between the young and aging rats. In aging rats, IXD extract at a dose of 100 mg/kg increased the total saliva significantly compared with younger rats in the same treatment condition (*p* < 0.05) (Figure 1B). The salivary flow rate relative to the SMG weight showed that aging rats had diminished saliva flow compared with young rats (*p* < 0.05). However, 50 mg/kg and 100 mg/kg of IXD extract resulted in a conspicuous increase in saliva secretion in aging rats (*p* < 0.05) (Figure 1C,D).

### 3.2. Morphological Analysis of Young and Aged Submandibular Gland Treated with Water or IXD Extract

**Figure 2:**
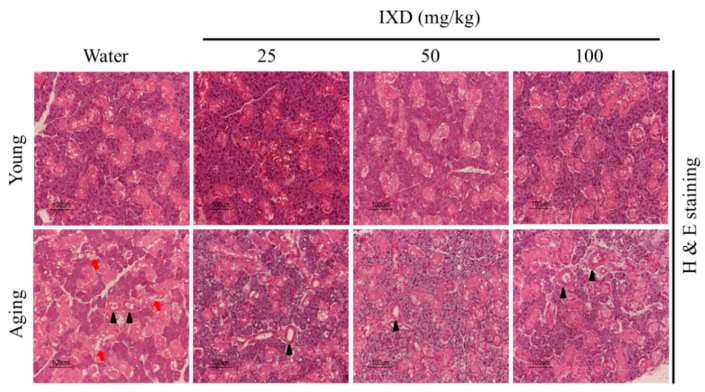
H and E staining was performed on formalin-fixed, paraffin-embedded submandibular gland sections to determine the morphological changes in water- and IXD-treated young and aging rats. Black arrowheads denote striated ducts, and red arrows indicate the acinar cells of the submandibular glands. Magnification: 400×, Scale bar: 100 µm.

The histological examinations revealed that the aging rats exhibited highly depleted acinar cells and dilated striated ducts of the submandibular glands, whereas the young rats had compact acinar cells and well maintained striated ducts. The morphology and numbers of acinar cells and striated ducts were well maintained in the aging groups that received 50 mg/kg and 100 mg/kg of IXD (Figure 2). These data suggest that IXD extract has the potential to increase saliva in aging rats and might relieve dry mouth symptoms.

### 3.3. Ixeris dentata Extract Increased the Alpha-Amylase Protein Expression and Its Activity in the Aged Submandibular Glands

**Figure 3:**
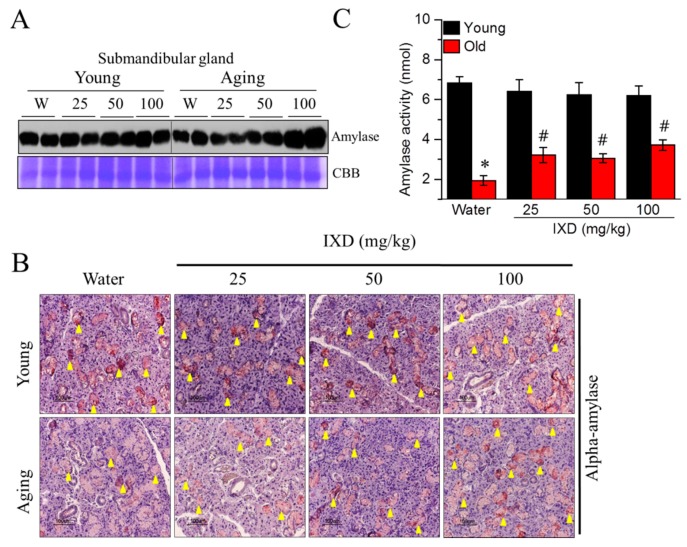
IXD extract increases alpha-amylase expression and water channel activation in aged submandibular glands. (**A**) Amylase protein expression, shown by western blot analysis; (**B**) Immunostaining was performed in submandibular glands using the alpha-amylase antibody. Yellow arrowheads indicate positive staining of alpha-amylase; Magnification: 400×, Scale bar: 100 µm; (**C**) Measurement of salivary amylase activity. Values are presented as mean ± SEM. * *p* < 0.05 vs. young control rats, # *p* < 0.05 vs. old control rats.

The expression and reactivity of amylase in the submandibular glands of aging rats were weak, and they increased following treatment with the IXD extract (Figure 3A,B). Moreover, 100 mg/kg of IXD extract led to strong, positive α-amylase expression in the SMG (Figure 3A) acinar cells (Figure 3B lower panel) of aging rats. Amylase expression was well maintained in the young rats treated with water or IXD (Figure 3A,B upper panel). Moreover, the decreased amylase activity in the saliva of aging rats was significantly improved following treatment with IXD extract (*p* < 0.05) (Figure 3C), suggesting that IXD might be a potential regulator of amylase secretion.

### 3.4. Ixeris dentata Extract Increased the Expression of Water Channel Protein from Aged Submandibular Glands

**Figure 4:**
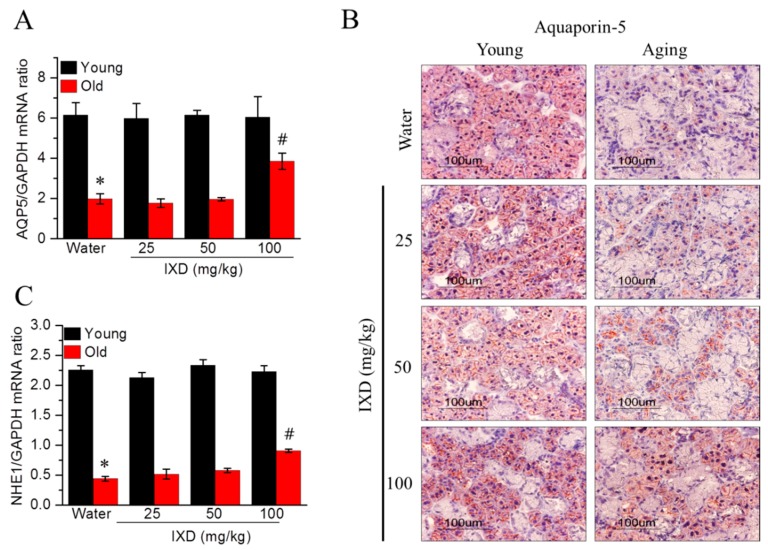
(**A**) Aquaporin-5 mRNA expression as evaluated by quantitative real-time PCR; (**B**) Aquaporin-5 protein expression as identified by immunostaining using the AQP5 antibody. Magnification: 400×, Scale bar: 100 µm; (**C**) Quantitative real-time PCR was performed to analyze NHE1 mRNA expression. Values are presented as mean ± SEM. * *p* < 0.05 vs. young control rats, # *p* < 0.05 vs. old control rats.

Because acinar cells are more responsible for water secretion than other cells, we examined AQP5 mRNA expression and localization in the submandibular sections of the indicated groups. As shown in Figure 4A,B, aging control rats showed a marked decrease in AQP5 mRNA (*p* < 0.05) and protein expression (acinar cell-specific) compared to young rats. The aging rats treated with 100 mg/kg of IXD extract showed drastically increased AQP5 expression in their submandibular glands (Figure 4A,B). As shown in Figure 4C, we observed that downregulated NHE1 mRNA, which causes deteriorating saliva secretion in aging rats, increased in response to IXD treatment (*p* < 0.05), suggesting that IXD could play a positive role in the fluid secretion mechanism. 

### 3.5. Acinar Cell-Specific Localization and Expression of IP3R2 and AQP5 Was Increased by the Treatment with IXD in Aging Submandibular Glands

**Figure 5:**
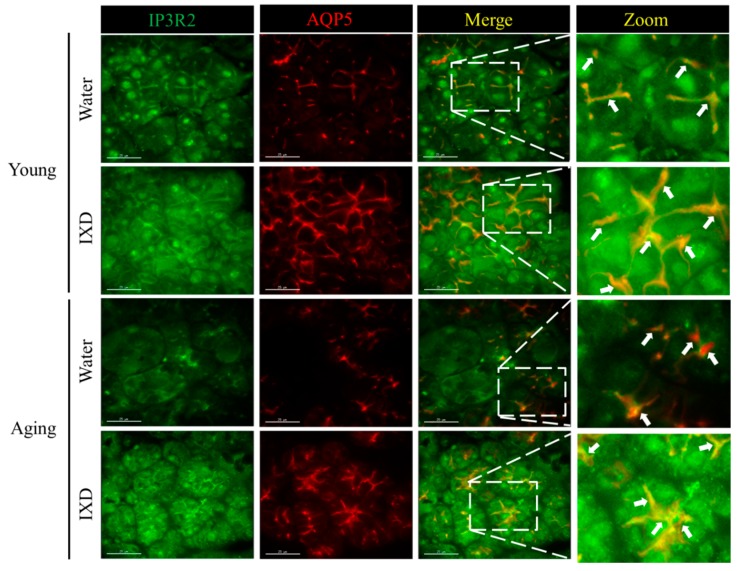
Expression of IP3R2 and Aquaporin-5 (AQP5) in submandibular glands from young and aging rats treated with water or IXD extract (100 mg/kg) was observed by confocal microscopy. Left panel shows the IP3R2 expression in young and aging rats treated as mentioned in the figure. Second panel show the AQP5 staining within apical region of the acinar cells. Third panel show the overlay of IP3R2 and AQP5. Right panel show the zoomed picture of the co-localized IP3R2 and AQP5. White arrows indicate the overlay of IP3R2 and AQP5 within apical regions of submandibular gland acinar cells. The young group shows a uniform and clear co-localization of IP3R2 and AQP5, aging control rats show less overlapping, and IXD treated aging rats show the increased expression and higher overlapping in the apical regions. Scale bar: 25 microns.

Inositol 1, 4, 5 triphosphate receptor (IP3R) in the ER is critical for mediating the release of ER calcium, increasing intracellular Ca^2+^ concentration, and allowing Ca^2+^ entry to the salivary gland acinar cells [28]. IP3R2 and IP3R3 are more responsible and participate in fluid secretion [6,28,29]. AQP5 is a well-known water channel protein as mentioned above. Here, we checked the expression of IP3R2 and AQP5 and demonstrated the colocalization pattern from the young and aging submandibular glands treated with water or IXD extract. We observed high expression of IP3R2 in the young rats specifically within the confines of apical regions of the acinar cells. The expression of IP3R2 in the apical region of acinar cells was attenuated in the aging rats and was activated by the treatment of IXD. Similarly, the expression and localization of AQP5 were observed in the apical portions of the acinar cells which were abundant in the young rats. The decreased expression of AQP5 in the aging submandibular glands was improved with the IXD treatment (Figure 5). Next, we examined the colocalization pattern of IP3R2 and AQP5 by using laser scanning confocal microscopy. As shown in Figure 5, young rats treated with water or IXD extract revealed uniform and higher overlapping of IP3R2 and AQP5 within the apical region of acinar cell premises. Aging control rats treated with water alone had lower expression and less overlapping of IP3R2 and AQP5. Interestingly, the expression and localization of IP3R2 and co-localization with AQP5 were restored by the IXD treatment in the apical region of acinar cells of aged submandibular glands (Figure 5). These results suggest that IXD may increase intracellular Ca^2+^ in the salivary glands cells (as evidenced in our previous article [25]) by activating IP3R2 (Figure 5). The increased intracellular Ca^2^^+^ triggers to increase the fluid and electrolyte secretion to the oral cavity via the AQP5 channel. 

### 3.6. Aging-Induced Free Radical Generation Was Attenuated by Ixeris dentata Extract

**Figure 6:**
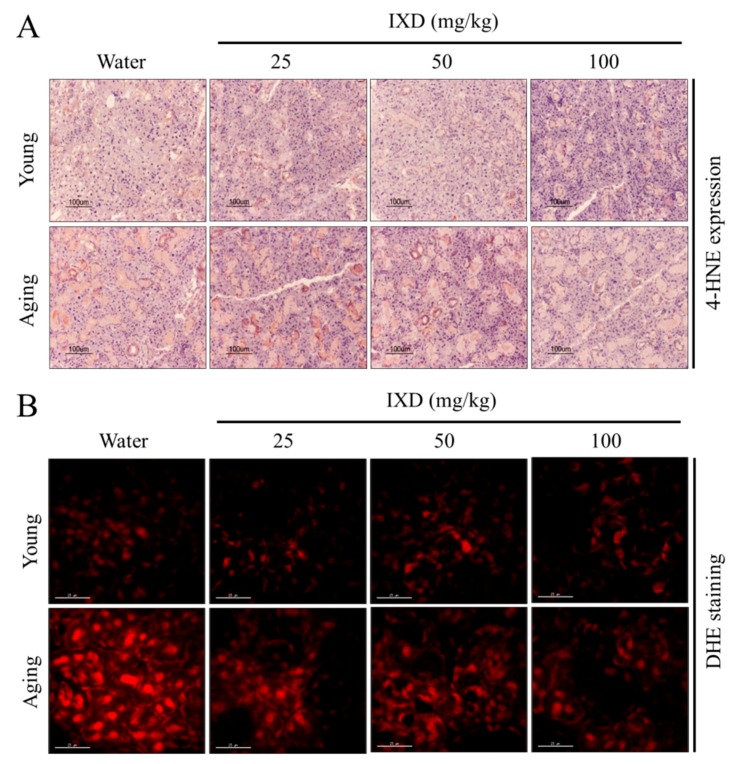
Aging-induced free radical generation was attenuated by IXD extracts. (**A**) Immunohistochemistry data revealing 4-HNE expression in the submandibular glands of the indicated groups. Magnification: 200×, Scale bar: 100 µm. (**B**) Dihydroethidium (DHE) fluorescent staining indicating reactive oxygen species (ROS) accumulation in the submandibular glands of the indicated rat groups. Pictures were taken using a tetramethylrhodamine isothiocyanate (TRITC) channel in confocal microscopy. Magnification: 600×, Scale bar: 25 µm.

Various reports have suggested that oxidative stress increases significantly with age [30,31]. We performed immunohistochemical staining for 4-HNE as a marker of oxidative stress, and we used the DHE fluorescent stain to detect ROS accumulation in the submandibular gland. As shown in Figure 6A, we observed high 4-HNE expression in the submandibular glands of aging rats, and IXD extract treatment dose-dependently reduced the expression of 4-HNE in aged SMG tissue. No differences in 4-HNE expression were observed in young rats treated with water or IXD extract (Figure 6A). Similarly, we observed high DHE fluorescence in the submandibular glands of aging rats, and IXD extract treatment reduced the ROS fluorescence intensity in a dose-dependent manner. There were no differences in young rats treated with water or IXD extract (Figure 6B). 

### 3.7. IXD Extract Regulates Disulfide Bond Formation during Oxidative Protein Folding

**Figure 7:**
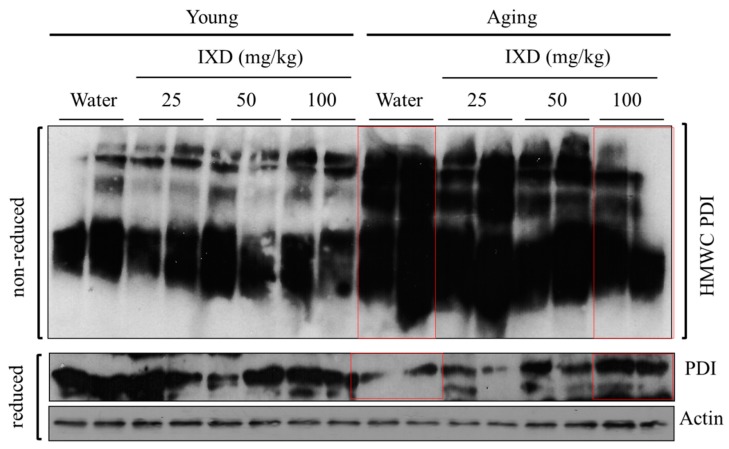
IXD extract regulates the formation of disulfide bonds during protein folding. Whole submandibular gland lysates from the indicated groups were analyzed for the presence of protein disulphide isomerase (PDI) in the high molecular weight complexes (HMWCs) on non-reducing (top panels) and reducing gels (bottom panels). PDI, Protein disulfide isomerase; HMWCs, high molecular weight complexes. Red lines highlight the expression of PDI in water and IXD treated aging submandibular glands lysate.

We evaluated the redox status of PDI in the submandibular glands of young and aging rats treated with water or IXD extract. We found hyper-oxidized PDI in aging rats by observing the PDI presence within the HMWC fractions (Figure 7, top panel), suggesting that highly oxidized PDI is associated with client proteins. The persistence of oxidized PDI in aging rats was alleviated by treatment with 50 and 100 mg/kg of IXD extract (Figure 7, top panel). Young animals treated with water or IXD extract showed less HMWC PDI than their aging counterparts (Figure 7, top panel). Similarly, we assessed the expression of PDI in the reducing gel and found decreased expression in the water-treated aging control rats. Because PDI improves oxidative protein folding capacity, decreased PDI expression might perturb the ER environment, causing misfolded proteins and leading to ER stress. Interestingly, IXD treatment at a dose of 50 or 100 mg/kg dramatically increased PDI expression, suggesting the potential role of IXD in ER homeostasis (Figure 7, bottom panel). 

### 3.8. IXD Extract Regulates Aging-Induced UPR Impairment in the Submandibular Glands

**Figure 8:**
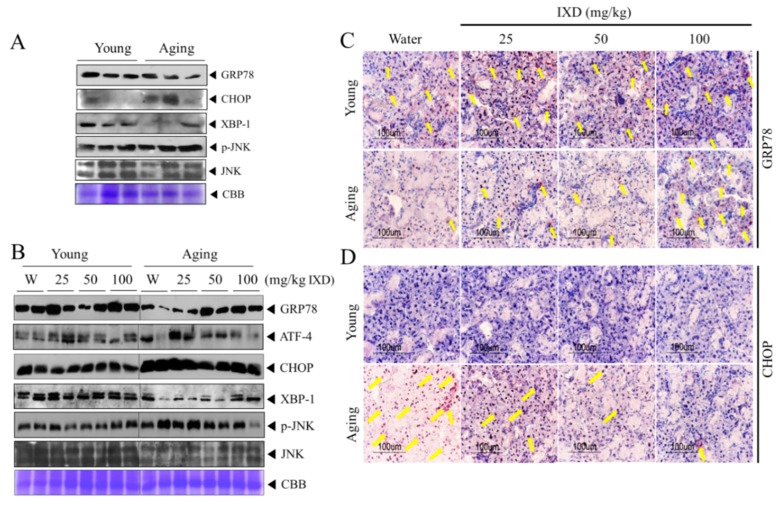
IXD extract regulates aging-induced unfolded protein response (UPR) impairment in the submandibular gland. (**A**) Western blotting was performed to demonstrate the UPR in young and aging submandibular glands. Protein expressions of the endoplasmic reticulum (ER) stress markers GRP78, CHOP, XBP1, and p-JNK was determined. CBB staining was done to show the equal loading pattern as a control. (**B**) The protein expression of different UPR components (GRP78, ATF-4, CHOP, sXBP1, p-JNK, and JNK) was analyzed in the submandibular glands of young and aging rats treated with water or IXD extract. (**C**,**D**) Immunolocalization of GRP78 and CHOP in the submandibular glands of the indicated groups. Magnification: 400×, Scale bar: 100 µm. UPR, Unfolded protein response; CBB, Coomassie brilliant blue. Yellow arrows indicate the positive expression of GRP78 and CHOP.

To understand the cellular mechanism for aging-induced diminished saliva secretion, we evaluated the ER stress response in the submandibular glands of young and aging rats. We observed a decline in 78 kDa-glucose regulated protein (GRP78), an ER chaperone, in the aging rats, whereas the young rats had plentiful expression of GRP78 (Figure 8A–C). C/EBP homologous protein (CHOP), a multifunctional transcription factor in the ER stress response with a role in inducing cell death by promoting protein synthesis and oxidation, occurred in much greater quantities in the aging rats than in the young rats (Figure 8A,B,D). On the other hand, X-box-binding protein 1 (XBP1), another unfolded protein response (UPR) component, decreased with aging. The expression of CHOP, phosphorylated c-Jun N-terminal kinase (p-JNK), and activating transcription factor-4 (ATF-4), which all participate in ER stress-mediated apoptosis signaling, increased greatly in the aging rats (Figure 8A,B). These data suggest that many of the proteins involved in cell survival or UPR decline over time, whereas pro-apoptotic signaling is activated during the aging process, which might deter salivary flow. In another set of experiments, we found that treatment with IXD extract increased the expression of GRP78 and XBP1 and decreased the expression of CHOP, ATF-4, and phosphorylated JNK in aging rats (Figure 8B). Likewise, the immunohistochemistry data revealed that IXD extract reversed the reduced acinar cell–localized GRP78 expression seen in the aging control rats (Figure 8C). The strong nuclear localization of CHOP in the submandibular glands of aging rats was downregulated with the application of IXD, suggesting that IXD might act as a regulator of ER stress (Figure 8D). There were no visible differences in the expression of GRP78 or CHOP among the treated and untreated young rats. In those ways, we demonstrated that ER stress is a possible mechanism for diminished saliva secretion with age, and that IXD might help maintain ER proteostasis and saliva secretion (Figure 8B–D).

## 4. Discussion

Age is one of the factors to make changes in the quality and quantity of saliva. For example, declined total salivary flow rate, increased viscosity, and changes in organic and inorganic salivary components [32] make a miserable life. The changes of saliva consequently decrease the quality of life by dysphagia, speech alteration, and increasing dental risk such as gingivitis, caries, periodontitis, and oral infections [11,25,33,34]. Age-associated periodontal disease involves inflammation and altered immune function and has been reported to be attenuated by the treatment with rapamycin [35], a potent mTOR inhibitor, known to inhibit inflammation and improve healthy lifespan [35,36,37]. Rapamycin has also been reported to increase salivation by around 46% and prevent hypo-functioning of salivary glands in irradiation-induced salivary hypofunction swine model [38]. Along with rapamycin, other treatments such as pilocarpine, cevimeline, and anethole trithione have been reported to increase saliva secretion and alleviate dry mouth syndrome [39]. Considering scaling and root planing, a conventional therapy, does not thoroughly remove periodontal pathogens, desiccant agent and diode laser therapy adjunct with scaling and root planing have been reported to reduce the inflammatory mediators in chronic periodontitis patients [40,41]. In addition, various natural medicines and compounds such as ginger herbal spray, red ginseng, and resveratrol have been practiced for the prevention of hyposalivation [6,42,43,44]. Our data demonstrated the cellular mechanism of aging-associated dry mouth and protection against it by the treatment with IXD extract.

Some animal studies have reported that salivary gland dysfunction is prevalent in aging animals. The salivary flow rate normalized to animal body weight was found to be significantly decreased in 90-week-old mice [45]. There is not a consistent report regarding the salivary flow rates of different age groups. One previous study reported that the stimulated salivary flow rate was significantly higher in the young group than in the middle-aged and elderly subjects [46]. This was confirmed in 48- and 72-week-old mice, whose stimulated whole salivary flow rate was significantly reduced compared with young control mice [47]. On the other hand, another report found that the stimulated salivary secretion was unaltered in aging rats, though age did affect the glandular morphology. However, the aging rat model for that study was only nine months old [48]. In our study, we used young and 20 months old rats to demonstrate the rate of salivary secretion. We observed that the weight of submandibular glands was significantly higher in aging rats as compared to their young counterparts. The total salivary secretion was unaltered in water-treated young and aging rats. Interestingly, salivary flow rate normalized to submandibular gland weight was significantly diminished in the aging rats and was further improved by IXD treatment (Figure 1C,D). In addition, we observed a reduced number of SMG acinar cells (which play a major role in saliva secretion [6]) in old animals (Figure 2, bottom panel), which suggests poor salivary gland functioning. None of the treated or untreated young rats showed reduced saliva secretion because they all had an abundant number of acinar and duct cells, and the morphology was well maintained. As indicated by our results, the aging rats exhibited very low acinar cell-localized AQP5 expression, suggesting a decrease in the water channel protein that influences salivation (Figure 4B). The increased expression of AQP5 and NHE1 in the salivary glands of aged rats treated with IXD (Figure 4A–C) suggests that IXD assists in both primary and secondary saliva secretion, which is consistent with our previous finding [21]. Increasing evidence suggests that NHE1 predominantly localizes to submandibular gland duct cells, where it regulates the flux of Cl^−^ and HCO_3_^−^ ions across acinar cells by sustaining the intracellular pH in both the stimulated and resting states [21,25,49]. In addition, IP3 has a key role in the secretion of salivary fluid [28] and tears [50]. IP3 binds with IP3R in the ER membrane and induces Ca^2+^ release from the ER to the cytosol via IP3R. Majorly, IP3R2 and IP3R3 are localized in the salivary glands and participate in fluid secretion. Previous studies reported that the mice lacking IP3Rs exhibit dry mouth by reducing salivary fluid secretion and dry eyes by limiting tear secretion [6,28,29,50]. Our previous study suggests that IXD has a promising potential to increase salivation in diabetes-associated xerostomia through increasing intracellular Ca^2+^ and activating AQP5 channel. Our current study demonstrates that aging attenuates the IP3R2 and AQP5 expression in the acinar cells, resulting in loss of salivary secretion. However, IXD activates and increase IP3R2 and AQP5 channel and ameliorate salivary flow from aging submandibular glands. The co-localization of IP3R2 and AQP5 shows the increased expression of both proteins in the apical regions of the salivary acinar cells (Figure 5). Because acinar cells are thought to have a greater role in fluid secretion and participates in primary saliva secretion. Likewise, NHE1 localize to the duct cells and modify primary saliva to generate the final fluid which is also called secondary fluid secretion [6,49]. Therefore, we evaluated the mRNA expression of AQP5 (localize to acinar cells) and NHE1 (duct cells localization) in the young and aging salivary gland and found that aging decreases the expression of AQP5 and NHE1 leading to salivary dysfunction. Surprisingly, treatment with IXD restored the expression of AQP5 and NHE1 in aging salivary glands (Figure 4A,C) subsequently enhanced salivary secretion. 

The excessive production of ROS contributes to salivary gland dysfunction, resulting in xerostomia. ROS has been suggested as a mechanism of salivary gland hypofunction in Sjogren’s syndrome [6]. A rapid decline in glutathione and increase in intracellular ROS suppressed the amylase release induced by a beta-adrenergic agonist in rat parotid acinar cells. Therefore, ROS-induced oxidative stress in salivary gland tissue induces an alteration in the secretory function and reduces salivary proteins [6,51]. Changes in the cellular structure of salivary glands prompt a decrease in saliva-secreting cells (up to a 40% reduction of acinar cells), which alters cellular function and results in hyposecretion [6]. Furthermore, PDI can catalyze or accelerate the formation, reduction, and isomerization of S-S bonds and bind to polypeptide chains [17]. A high accumulation or aggregation of proteins has been observed in previous studies. Notably, we observed HMWC PDI in the aging rats (Figure 7), revealing the aggregation or accumulation of the client proteins that cause impairment in the ER, leading to misfolded proteins. The continuation of HMWC could lead to the disposition of insoluble multiprotein complexes that eventually disturb ER function and induce apoptosis. The abnormal oxidation of PDI in aging might be one of the factors that generate ROS and increases the ER stress response. The overproduction of ROS alters the redox status, just as hyper-oxidized PDI does, which increases oxidative and ER stress.

Increased oxidative stress and reduced stress tolerance accompany chronological aging and lead to oxidative injury. This could reduce the pro-survival signals in response to stress or activate ER stress, which is linked to highly expressed proapoptotic GADD153/CHOP and JNK activation [52,53,54]. Under aging-induced chronic ER stress, the UPR adaptive responses decline and the increased oxidative stress and upregulation of several pro-apoptotic components (e.g., CHOP) induce cellular apoptosis [55]. A previous report stated that the continuation of an age-related declined defense system against unfolded proteins leads to an increase in protein ubiquitination and a decrease in chaperones such as PDI, GRP78, and calnexin [56]. Similarly, another study revealed the age-associated reduction of PDI and GRP78 activity and has been correlated with increased protein carbonylation in the liver tissue [57]. Our previous study suggested that the increased protein carbonylation and decreased PDI expression is one of the mechanisms of diabetes-associated xerostomia [21]. Our current study illustrates the HMWC of PDI indicating a high level of protein aggregation and lower expression of PDI in the aging submandibular glands (Figure 7). The loss of PDI and GRP78 which have a role in chaperone activities, may increase age-dependent misfolded proteins and display aging characteristics. Our data indicate that IXD improve salivary gland functions by reducing HMWC of PDI and increase protein expression of GRP78 and PDI in the aging submandibular glands. 

The apoptotic pathway, with the indication of highly expressed CHOP, was suggested as a flawed UPR in aged rat hippocampus [56]. CHOP promotes ER stress-induced apoptosis, supporting that older animals are more responsive than younger ones to cellular apoptosis aroused by protein accumulation [56]. During our study, reduced expression of the ER chaperone GRP78 in aged submandibular glands impaired the ER folding environment, and IXD displayed an ability to restore ER homeostasis (Figure 8A–C). We observed high expression of ATF-4, CHOP, and p-JNK in the submandibular glands of aging rats (Figure 8A,B), suggesting that an increase in the ER stress response in aging negatively influences salivary secretion, which could be improved by IXD treatment (Figure 1C). This report and other previous studies suggest that the increased apoptosis associated with the aging process leads to diminished saliva secretion [45,47]. Our data suggest that disruption in the ER environment resulting from aging could lead to hypo-functioning of the salivary glands, and that IXD extract could improve their functioning.

## 5. Conclusions

**Figure 9:**
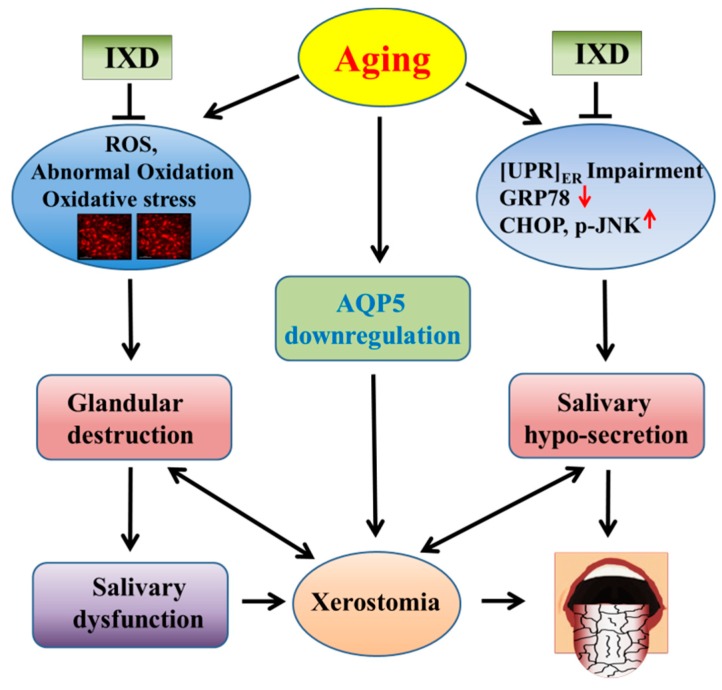
The proposed mechanism of an IXD extract against aging-triggered dry mouth. This graphic representation shows the mechanism of aging-induced xerostomia and the application of IXD extract against aging-associated dry mouth syndrome. This figure shows the aging-associated abnormal oxidation of proteins, increased oxidative stress, impairment of the UPR components (decreased GRP78 and increased CHOP and JNK), decreased water channel protein AQP5, leading to salivary gland destruction and hypo-secretion. This may cause salivary gland dysfunction and deters salivary flow leading to dry mouth syndrome. The graphic shows that IXD extract has a potential to regulate aging-associated ER stress and oxidative stress, increase AQP5, increase salivary secretion and comfort from xerostomia.

The main focus of our study was to investigate the medications which can stimulate the salivary glands and increase salivary secretion to improve the aging-associated dry mouth sensation. The key finding of this study suggests that the IXD extract has a potential to improve salivary flow from aging rats by modulating oxidative stress and UPR as illustrated in Figure 9. IXD may be beneficial to the geriatric patients who suffer from hyposalivation, xerostomia, or aging-associated hyposalivation-induced periodontal diseases. Further clinical trial is required to confirm the beneficial effect of IXD in aging-associated xerostomic patients.
